# Whole-Genomic and Functional Characterization of *Lactiplantibacillus plantarum CLPX21* as Potent Probiotic Candidate

**DOI:** 10.3390/microorganisms14081789

**Published:** 2026-08-14

**Authors:** Shichao Xu, Qianqian Fan, Hongdou Liu, Yilin Lu, Yuqian Liu, Zhouyuan Wang, Yunxia Li, Rendong Fang, Zhiwei Li, Lianci Peng

**Affiliations:** 1 Joint International Research Laboratory of Animal Health and Animal Food Safety, College of Veterinary Medicine, Southwest University, Chongqing 400715, China; jjepic@163.com (S.X.); 15212301956@163.com (Q.F.); lhd3040461836@163.com (H.L.); liuyuqian626@163.com (Y.L.); liyunxia2025@swu.edu.cn (Y.L.); rdfang@swu.edu.cn (R.F.); 2 Kunming Hemeihua Feed Limited Company, Kunming 682100, China; 18182335744@163.com

**Keywords:** *Lactiplantibacillus plantarum*, probiotic properties, antibacterial activity, anti-inflammatory activity, bacteriocin

## Abstract

Lactic acid bacteria (LAB) are regarded as promising probiotics with multiple beneficial properties for humans’ and animals’ health. In this study, ten LAB isolates were screened and *Lactiplantibacillus plantarum* (*L. plantarum*) CLPX21 was selected as a candidate strain with its probiotic potential based on *in vitro* screening of antimicrobial activity and environmental stress tolerance, including its resistance to acidic environments and bile salts. This strain displayed adhesion capacity with an auto-aggregation rate of 47.15% at 24 h, co-aggregation rates above 67% with pathogenic bacteria, and an ability to adhere to IPEC-J2 cells (6.37%). CLPX21 showed broad-spectrum antimicrobial activity against common foodborne pathogens including *Escherichia coli* (*E. coli*), *Salmonella*, and *Staphylococcus aureus*. In addition, CLPX21 inhibited *E. coli* biofilm formation. Furthermore, CLPX21 significantly suppressed *E. coli*-induced inflammatory cytokine production in mouse peritoneal macrophages. Importantly, CLPX21 did not exhibit hemolytic activity. Genomic analysis further revealed that the CLPX21 genome encoded multiple functional genes and gene clusters, including biosynthesis of bacteriocins and secondary metabolites associated with antimicrobial and antioxidant functions, which provide a genetic basis for its beneficial phenotypic characteristics. In conclusion, *L. plantarum* CLPX21 displays probiotic properties with potent antimicrobial and anti-inflammatory activities, representing a promising candidate strain for applications in the food and health industries.

## 1. Introduction

Foodborne pathogens such as *Staphylococcus aureus* (*S. aureus*), *Escherichia coli* (*E. coli*), and *Salmonella* opportunistically exist in various foods, including meat, eggs, and dairy products, causing severe foodborne illnesses in humans which threaten global public health and food safety [1]. These foodborne bacteria cause diarrheal and invasive infectious disease, leading to significant morbidity and mortality. Notably, foodborne bacteria-associated antimicrobial resistance (AMR) has been increasingly implicated as a major global public health concern [2], with the World Health Organization (WHO) declaring it as one of the top ten current societal challenges. Importantly, these foodborne bacteria can form biofilms, which greatly exacerbate the risk of foodborne diseases. Biofilms not only enhance bacterial tolerance to adverse environmental stress, but also serve as key hubs for horizontal gene transfer of AMR [3]. To address these challenges, probiotics, as eco-friendly and effective species, have drawn more attention to combating foodborne pathogens due to their antimicrobial and immunomodulatory activities.

Lactic acid bacteria (LAB) are widely present in food, the environment, and animal intestines, and fermented foods are considered as the primary ecological source for LAB [4]. *Lactiplantibacillus plantarum* (*L. plantarum*) is widely recognized as a promising probiotic due to its strong tolerance to gastrointestinal fluids, thermal stability, robust intestinal colonization capacity, and rich antimicrobial metabolites. In addition to extensive antimicrobial activity [5,6,7], *L. plantarum* isolated from various sources has been reported to alleviate intestinal inflammation through maintaining intestinal barrier integrity and inhibiting inflammatory cytokine production [8,9,10]. Therefore, developing probiotics as additives is a promising strategy to alleviate infectious and non-infectious diseases [11].

In this study, 10 LAB strains were isolated from fermented vegetables and screened for their antimicrobial activity and stress tolerance properties. Among these isolates, *L. plantarum* CLPX21 was selected as the most promising candidate strain based on its superior performance in the initial screening. We further characterized its genomic and functional properties. CLPX21 exhibited potent antimicrobial and anti-inflammatory activities. Our study provides new insights for the discovery and potential application of functional probiotics.

## 2. Materials and Methods

### 2.1. Bacterial Strains and Culture Conditions

LAB strains were isolated from fermented vegetables (spicy sour cabbage from Hengxi Town, Zhejiang Province, China) and cultured at 37 °C under aerobic conditions on Man–Rogosa–Sharpe (MRS; Oxoid Inc., Pittsburgh, PA, USA) agar plates or in MRS broth. Pure cultures were isolated using the spread-plate method on MRS agar, and a single colony was picked and streaked repeatedly to obtain pure isolates. These LAB strains were stored at −80 °C in MRS medium containing 25% glycerol.

Pathogenic bacterial strain *E. coli* (RS218) was obtained from Professor Xiangru Wang at the College of Veterinary Medicine, Huazhong Agricultural University, Wuhan, China. *E.* *coli* MCA1 and L2 were isolated from infected piglets. *S. aureus* (NCTC-8325) was purchased from BioVector NTCC Inc. (Beijing, China). *Salmonella* was isolated from lungs of calves and obtained from Associate Professor Fang He at the College of Veterinary Medicine, Southwest University, Chongqing, China. These strains were stored at −80 °C and cultured at 37 °C on LB (Luria–Bertani; Aobox, Beijing, China) plates or in LB broth. These stored pathogenic bacteria were grown on LB plates at 37 °C for 18–24 h, following which a single colony was inoculated into 10 mL of LB broth and cultured at 37 °C for 4–5 h until the bacterial culture reached the logarithmic growth phase. Subsequently, these bacterial suspensions were diluted to a concentration of 1 × 10^6^ CFU/mL and used for the assays described below.

### 2.2. The Detection of Antimicrobial Activity of LAB

The antibacterial activity of cell-free filtrate (CFF) from LAB was quantitatively assessed using the micro-broth dilution method. LAB were incubated in MRS broth at 37 °C for 24 h. The culture was then centrifuged at 1920× *g* at 4 °C for 15 min to collect the supernatant, which was sterile-filtered through a 0.22 μm membrane filter to prepare the CFF sample for subsequent assays. Next, 100 μL of the CFF was two-fold diluted with LB broth (ranging from 50% to 25%) and then mixed with an equal volume of the pathogenic bacterial suspension (1 × 10^6^ CFU/mL) in 96-well plates, resulting in final concentrations of CFF of 25% and 12.5% (*v*/*v*). After incubation at 37 °C for 18 h, the minimum inhibitory percentage (MIP) was defined as the lowest percentage of CFF with no visible bacterial growth. Next, 100 μL of mixed culture from wells without visible bacterial growth were incubated on LB agar plates at 37 °C overnight to determine the minimum bactericidal percentage (MBP) as the lowest percentage of CFF without bacterial growth on LB agar plates.

For the determination of the antibacterial activity of the whole-cell suspension (WCS) of LAB, LAB were incubated in MRS medium at 37 °C for 24 h. The culture was then centrifuged at 1920× *g* at 4 °C for 15 min to collect a bacterial pellet, which was subsequently resuspended in MRS broth. Next, 100 μL of WCS was mixed with an equal volume of pathogen suspension (1 × 10^6^ CFU/mL) in a 96-well plate, resulting in final WCS concentrations of 25% and 12.5% (*v*/*v*). After incubation at 37 °C for 18 h, 100 μL of mixed culture was incubated on LB agar plates at 37 °C overnight to determine the MBP of the WCS as the lowest percentage of WCS without bacterial growth on LB agar plates.

### 2.3. Tolerance to Acid and Bile Salts of LAB

To determine tolerance to acid, LAB were overnight-cultured and LAB suspensions were then diluted with fresh MRS broth to adjust the *A*_600_ to 1.0, followed by subculture at a 1:5 ratio with MRS broth at different pH levels (2, 3, 4, and 6.2) at 37 °C, allowing sufficient room for growth. In this study, *A*_600_ was used as a rapid screening tool to compare the relative growth of 10 LAB strains under different stress conditions. Growth was measured as an increase in *A*_600_, reflecting the increase in LAB cell number from growth. After 4 h of incubation, the *A*_600_ absorbance of the bacterial suspension was measured. The LAB survival rate at pH = 6.2 was defined as the control. The formula for calculating the survival rate (%) of LAB is as follows:

$$=(A_{pHsample}/A_{pH6.2})\times 100\%:$$

where *A*_pHsample_ is the *A*_600_ value after 4 h of incubation at the respective pH, and *A*_pH6.2_ is the *A*_600_ value after 4 h of incubation at pH 6.2.

To determine tolerance to bile salts, overnight-cultured LAB suspensions were diluted with fresh MRS broth to adjust the *A*_600_ to 1.0, followed by subculture at a 1:5 ratio with MRS broth in the presence or absence of different concentrations of bovine bile salt (0.1%, 0.2%, and 0.3%) at 37 °C. After 4 h of incubation, the *A*_600_ absorbance of the LAB suspension was measured. The *A*_600_ value of LAB without bovine bile salt was defined as the control. The formula for calculating the survival rate (%) of LAB is as follows:

$$=(A_{sample}/A_{control})\times 100\%.$$

where *A*_sample_ is the *A*_600_ value after 4 h of incubation with bile salt, and *A*_control_ is the *A*_600_ value after 4 h of incubation without bile salt.

### 2.4. The Detection of Growth Curve of LAB

LAB strains were firstly cultured in MRS medium at 37 °C for 18 h, following which a 50 μL LAB suspension was inoculated into 20 mL of MRS broth and cultured at 37 °C for the indicated times. Next, the *A*_600_ absorbances of the LAB suspensions were measured at 0, 2, 4, 5, 6, 7, 8, 10, 12, 14, 16, 22, and 24 h post-incubation.

### 2.5. In Vitro Adhesion Ability

Auto-aggregation of CLPX21 was induced using the method from Kumari et al. [12]. In short, LAB were cultured in MRS broth as described above. The cells were then harvested and resuspended in PBS to a concentration of 1 × 10^9^ CFU/mL. The suspension was incubated at 37 °C, and samples were taken at 0, 2, 4, 6, 8, and 24 h. At each time point, the upper solution was carefully aspirated, and its absorbance was measured at 600 nm. The auto-aggregation rate (%) was calculated using the formula $((A0-A_{sample})/A0)\times 100\%$.

The absorbance of the upper solution at 0 h was defined as A0.

For the co-aggregation determination, LAB suspension (1 × 10^9^ CFU/mL) was mixed with an equal volume of pathogenic bacterial suspension (*E. coli*, *S. aureus*, and *Salmonella*) and incubated at 37 °C for 0 and 2 h. After mixing, the supernatant was aspirated, and its absorbance was measured at 600 nm [13]. The formula for calculating the co-aggregation rate (%) was as follows: $\left(1-Amix/\left(A1+A2\right)/2\right)\times 100\%$. The absorbances of the upper solution of the LAB alone and pathogenic bacteria alone at 0 h were defined as *A*1 and *A*2, respectively. The absorbance of the upper solution of the LAB mixture at 2 h was defined as *A*mix.

For the in vitro adhesion assay, porcine intestinal epithelial IPEC-J2 cells were grown in DMEM supplemented with 10% FBS at 37 °C with 5% CO_2_ until they reached 90% confluence. Then, cells were digested by 0.01% trypsin–EDTA and seeded into 12-well plates at 1 × 10^5^ cells/well for overnight incubation. Next, LAB suspension (5 × 10^6^ CFU) in DMEM containing 10% FBS was added to the cells, and was then incubated for 2 h at 37 °C with 5% CO_2_. Following incubation, cells were washed with PBS and then lysed with 0.1% Triton X-100 for 15 min. Adherent LAB were counted on MRS plates as N_adhesion_ and the adhesion rate was calculated as follows: adhesion rate (%) =$(N_{adhesion}/N0)\times 100\%$. The initial number of LAB was defined as N_0_.

### 2.6. Time–Killing Curves of LAB

Firstly, LAB in the logarithmic growth phase were diluted in MRS broth to concentrations of 10^7^, 10^8^ and 10^9^ CFU/mL to prepare CFFs from LAB at these different concentrations. Then, 100 μL of CFF was mixed with an equal volume of *E. coli* RS218 (1 × 10^6^ CFU/mL) in 96-well plates and incubated at 37 °C for 0, 3, 6, 9, 15, 18, 21 and 24 h, respectively. To investigate the effect of acid on *E. coli* growth, untreated *E. coli* suspension was mixed with MRS broth at pH = 4. Untreated *E. coli* mixed with MRS broth at pH = 7.2 served as a control. After incubation for the indicated time, the *A*_600_ absorbance was measured. Meanwhile, after 15 h of incubation, 100 μL of mixed culture was ten-fold diluted and plated in LB agar plates for overnight culture at 37 °C, followed by colony counting. The number of colonies was defined as N. Growth inhibition rates were calculated as follows: $(\left(N_{control}-N_{sample}\right)/N_{control})\times 100\%$.

### 2.7. Anti-Biofilm Activity of LAB

*E. coli* RS218 suspension (1 × 10^7^ CFU/mL) was added into 48-well plates and then mixed with CFFs derived from LAB at different concentrations (10^7^, 10^8^ and 10^9^ CFU/mL), followed by incubation at 37 °C for 24 h. After incubation, supernatants were removed and biofilms were gently washed with PBS three times to remove free-floating bacteria. Then, crystal violet solution (0.1%) was added to stain the biofilms for 30 min. After these washing steps, 30% acetic acid was added to dissolve the biofilms. Finally, biofilm mass was determined at an absorbance of 570 nm.

### 2.8. Cytotoxicity Assay

Wild-type C57BL/6 mice (female, 8 weeks of age) were purchased from Chongqing Ensiwei’er Biotechnology Co., Ltd. (Chongqing, China). All mice were maintained under Specific Pathogen-Free (SPF) conditions. Mice were anesthetized to collect primary peritoneal macrophages. Peritoneal macrophages were collected by peritoneal lavage with RPMI1640 medium to harvest the cells for subsequent cytotoxicity assays and the detection of cytokine production. All animal experiments were approved by the Institutional Animal Care and Use Committee (IACUC) of Southwest University, Chongqing, China (permission No. IACUC-20241210-01). Briefly, the cells (1 × 10^6^ cells/mL, 200 μL) were seeded in 48-well plates and cultured in RPMI1640 medium supplemented with 10% FBS at 37 °C in a humidified incubator with 5% CO_2_ to assess the cytotoxicity of LAB using the trypan blue exclusion assay. LAB suspension (10^8^ CFU/mL) in the logarithmic growth phase was directly used to prepare CFF. Meanwhile, logarithmic-phase LAB were centrifugated and suspended in fresh RPMI1640 medium, resulting in a final concentration of 10^8^ CFU/mL, and were defined as activation LAB. In addition, a logarithmic-phase LAB suspension (10^8^ CFU/mL) was heated in a water bath at 85 °C for 15 min, followed by centrifugation and resuspension in RPMI1640 medium, with these bacteria defined as inactivation LAB. Subsequently, these different samples derived from LAB were added to cells and incubated at 37 °C for 24 h. After incubation, the cells were washed once with PBS and detached with 0.25% (*w*/*v*) trypsin and 0.53 mM EDTA for 2 min. Trypsin was then inhibited with pre-warmed RPMI 1640 medium containing 10% FBS. The cells were collected into sterile tubes and diluted with trypan blue (1:1). Live cells were counted microscopically using a hemocytometer, and the percentage of viable cells was calculated. Untreated cells were used as a control. The percentage of viable cells was calculated as follows: cell viability (%) = (sample/mock)×100%.

### 2.9. Hemolytic Evaluation

We used *S. aureus* as a positive control. CLPX21 was plated onto Columbia blood agar plates (BKMAM Biotechnology Co., Ltd., Changde, China) and incubated at 37 °C for 48 h to check for hemolysis around the colonies.

### 2.10. Enzyme Linked Immunosorbent Assay (ELISA)

Mouse primary peritoneal macrophages (1 × 10^6^ cells/mL, 200 μL) were prepared in 48-well plates. After cell attachment, cells were pretreated with different samples derived from LAB as described above at 37 °C for 24 h. After pretreatment, cells were washed with PBS and infected with *E. coli* RS218 at a multiplicity of infection (MOI) of 10 at 37 °C for 2 h. After 2 h of infection, gentamicin was added and cells continued incubation for 22 h. Finally, supernatants were collected and inflammatory cytokines including IL-1β and IL-6 were determined by ELISA according to the manufacturer’s instructions. The ELISA kits were purchased from Invitrogen (Carlsbad, CA, USA).

### 2.11. 16S rRNA Gene Sequencing

The genomic DNA of isolated LAB strains was extracted by a bacterial genomic DNA extraction kit (Sangon Biotech Co., Ltd., Shanghai, China). Primers for PCR amplification of the 16S rDNA were used as follows: 27F (5′AGAGTTTGATCCTGG CTCAG3′) and 1492R (5′GGTTACCTTGTTACGACTT3′). The amplified PCR products were sequenced and compared using the online NCBI BLAST tool (https://blast.ncbi.nlm.nih.gov/Blast.cgi, accessed on 11 October 2025). Multiple sequence alignment of 16S rRNA gene sequences was performed using ClustalW implemented in MEGA7 (version 7.0.26). A phylogenetic tree was constructed using the Neighbor-Joining (NJ) method, with branch reliability assessed through 1000 bootstrap replicates.

### 2.12. Whole-Genome Sequencing

Genomic DNA from isolated LAB strains was extracted using the Invitrogen PureLink^®^ Genomic DNA Kit according to the manufacturer’s instructions. The purified genomic DNA was then sequenced using the Illumina HiSeq2000 platform (Biozeron, Shanghai, China). Genome assembly was performed using Unicycler (https://github.com/rrwick/Unicycler, accessed on 15 October 2025). Genome annotation was conducted using various functional databases, including the Non-Redundant (NR) database in the NCBI, SwissProt (http://uniprot.org, accessed on 15 October 2025), KEGG (http://www.genome.jp/kegg/, accessed on 15 October 2025), GO (http://www.geneontology.org/, accessed on 15 October 2025), COG (http://www.ncbi.nlm.nih.gov/COG, accessed on 15 October 2025), CAZy (http://www.cazy.org/, accessed on 16 October 2025), CARD (https://card.mcmaster.ca/, accessed on 16 October 2025), PHI, TCDB (http://www.tcdb.org/, accessed on 16 October 2025), VFDB, BacMet, SignalP (http://www.cbs.dtu.dk/services/SignalP/, accessed on 16 October 2025), and TMHMM (http://www.cbs.dtu.dk/services/TMHMM/, accessed on 17 October 2025). BAGEL5 (http://bagel5.molgenrug.nl/, accessed on 17 October 2025) and antiSMASH analysis (https://antismash.secondarymetabolites.org/, accessed on 17 October 2025) were employed to identify gene clusters of bacteriocin biosynthesis and secondary metabolite biosynthesis, respectively.

### 2.13. Statistical Analysis

Statistical analysis was performed using GraphPad Prism 10.3 software (San Diego, CA, USA). All data are expressed as mean ± standard deviation (SD). Each experiment was performed independently three times with replicate samples for each group. Comparisons between two groups were conducted using the unpaired Student *t*-test, while comparisons among multiple groups were performed using one-way analysis of variance (ANOVA) followed by Tukey’s post hoc test. Exact *p* values for group comparisons are presented in the figures rather than using asterisk annotations. A value of *p* < 0.05 was considered statistically significant.

## 3. Results

### 3.1. Screening of LAB with Antimicrobial Activity and Probiotic Properties

#### 3.1.1. Antimicrobial Activity of LAB

**Figure 1:**
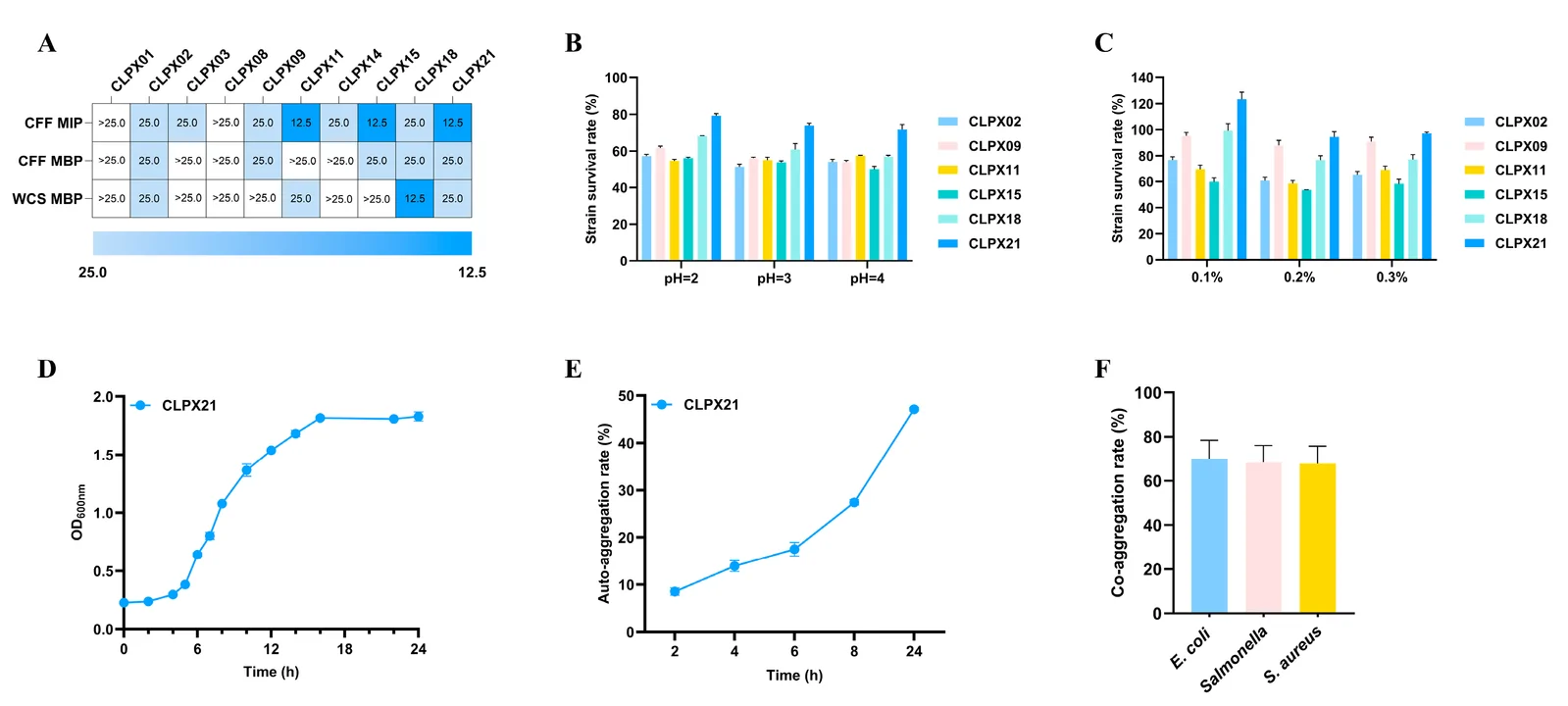
Screening of LAB with antimicrobial activity and probiotic properties. (**A**) MIP and MBP of CFFs and WCSs from 10 LAB strains against *E. coli* RS218. (**B**) Survival rate of LAB strains at pH 2.0, 3.0, and 4.0. (**C**) Survival rate of LAB strains at 0.1%, 0.2% and 0.3% bile salt. (**D**) Growth of CLPX21. (**E**) Auto-aggregation rate. (**F**) Co-aggregation with pathogenic bacteria.

To screen the potential probiotic LAB strains, we isolated 10 LAB strains from fermented vegetables and investigated their antimicrobial activity and probiotic properties. Among the 10 LAB strains, the CFF of 8 strains including CLPX02, CLPX03, CLPX09, CLPX11, CLPX14, CLPX15, CLPX18, and CLPX21 exhibited antibacterial activity to some extent against *E. coli* RS218, in which their MIP was 12.5% (*v*/*v*) or 25% (*v*/*v*) (Figure 1A). The MBP of CFFs from LAB strains including CLPX02, CLPX09, CLPX15, CLPX18, and CLPX21 was 25% (*v*/*v*) (Figure 1A). However, only the WCS of CLPX02, CLPX21, CLPX11, and CLPX18 exhibited their MBP at 12.5% (*v*/*v*) or 25% (*v*/*v*) (Figure 1A).

#### 3.1.2. Tolerance to Acid and Bile Salt of LAB

We evaluated the probiotic properties of LAB strains including CLPX02, CLPX09, CLPX11, CLPX15, CLPX18 and CLPX21, which exhibited the detachment of both MIP and MBP from CFF, or of MBP from WCS. The results showed that only CLPX21 exhibited high survival rate at pH 2.0, 3.0 and 4.0, with a survival rate ranging from 71.77% to 79.19% under each of these acidic conditions (Figure 1B). In addition, CLPX21 also exhibited robust tolerance to bile salt in vitro, with a survival rate ranging from 94.43% to 123.37% at 0.1%, 0.2% and 0.3% bile salt (Figure 1C). Based on these screening results, including antimicrobial activity, acid tolerance, and bile salt tolerance, CLPX21 was selected as the most promising candidate strain for further in-depth characterization.

#### 3.1.3. Growth Curve of LAB

To further investigate the basic function of CLPX21, we firstly determined its growth properties. During a 24 h culture period, CLPX21 remained in the lag phase from 0 to 4 h, the logarithmic phase from 4 to 16 h, and the stationary phase from 16 to 24 h (Figure 1D).

#### 3.1.4. Adhesion Ability of CLPX21

Bacterial aggregation ability plays an important role in promoting adhesion and biofilm formation [14]. The auto-aggregation ability enables probiotics to adhere to intestinal epithelial cells and establish colonization to exert their beneficial effects, while co-aggregation with pathogenic bacteria can inhibit the attachment of pathogens to intestinal epithelial cells and facilitate their elimination. In this study, the auto-aggregation rate of CLPX21 gradually increased with incubation time, reaching 47.15% at 24 h (Figure 1E). Regarding co-aggregation, CLPX21 showed high co-aggregation rates with *E. coli*, *Salmonella*, and *S. aureus* after 2 h, reaching 70.14%, 68.43%, and 67.77%, respectively (Figure 1F). In addition, the adhesion rate of CLPX21 to porcine intestinal IPEC-J2 cells was 6.37% (Table S1). These results demonstrate that CLPX21 possesses probiotic potential with considerable adhesion ability.

### 3.2. Antibacterial and Anti-Biofilm Activity of CLPX21

**Figure 2:**
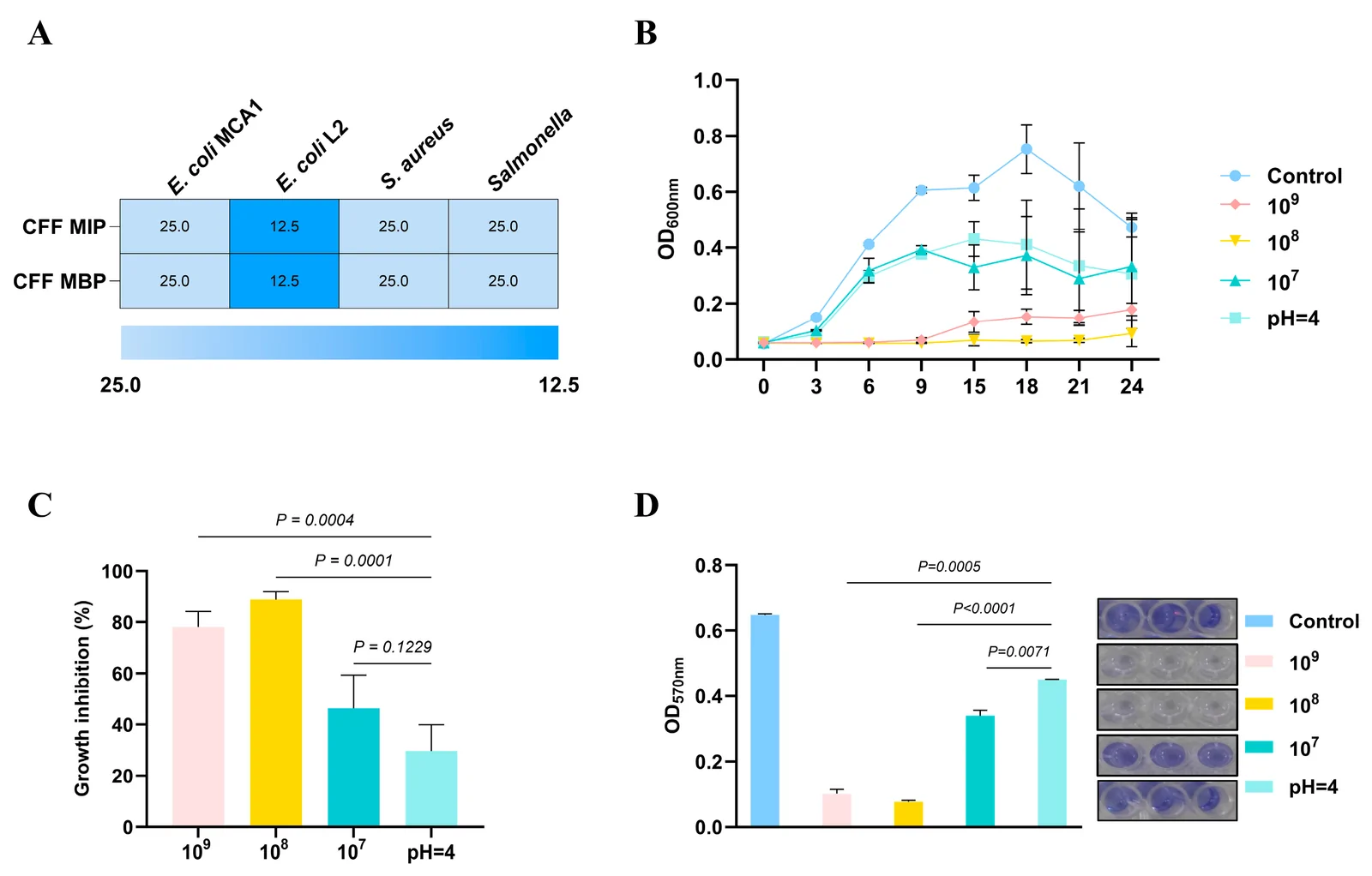
Antibacterial and anti-biofilm activity of CFF from CLPX21. (**A**) MIP and MBP of CFF from CLPX21 against pathogenic bacteria including *E. coli*, *S. aureus*, and *Salmonella*. (**B**) Time–killing curves of CLPX21 CFF at different concentrations against *E. coli* RS218. (**C**) Growth inhibition rates of CFF from CLPX21 at different concentrations after 15 h treatment against *E. coli* RS218. (**D**) Anti-biofilm activity of CFF from CLPX21 at different concentrations against *E. coli* RS218.

We further investigated whether CLPX21 exhibits antibacterial activity against other foodborne bacteria including *E. coli* MCA1, *E. coli* L2, *S. aureus* and *Salmonella*. The CFF from CLPX21 exhibited potent antibacterial activity against these pathogenic bacterial strains, with its MIP and MBP at 12.5% (*v*/*v*) or 25% (*v*/*v*) (Figure 2A). Furthermore, the results of antimicrobial activity at different concentrations of CFF from CLPX21 showed that CFF at concentrations of 10^8^ CFU/mL and 10^9^ CFU/mL effectively inhibited the growth of *E. coli* RS218 (Figure 2B). After 15 h of treatment, the inhibition rates of CFF from CLPX21 at concentrations of 10^9^, 10^8^, and 10^7^ CFU/mL were 78.1%, 88.7%, and 46.4%, respectively (Figure 2C). Notably, CFF from CLPX21 at concentrations of 10^9^, 10^8^, and 10^7^ CFU/mL significantly inhibited biofilm formation in a concentration-dependent manner (Figure 2D). These results demonstrate that CLPX21 exhibits potent antibacterial and anti-biofilm activity.

### 3.3. Anti-Inflammatory Activity of CLPX21

**Figure 3:**
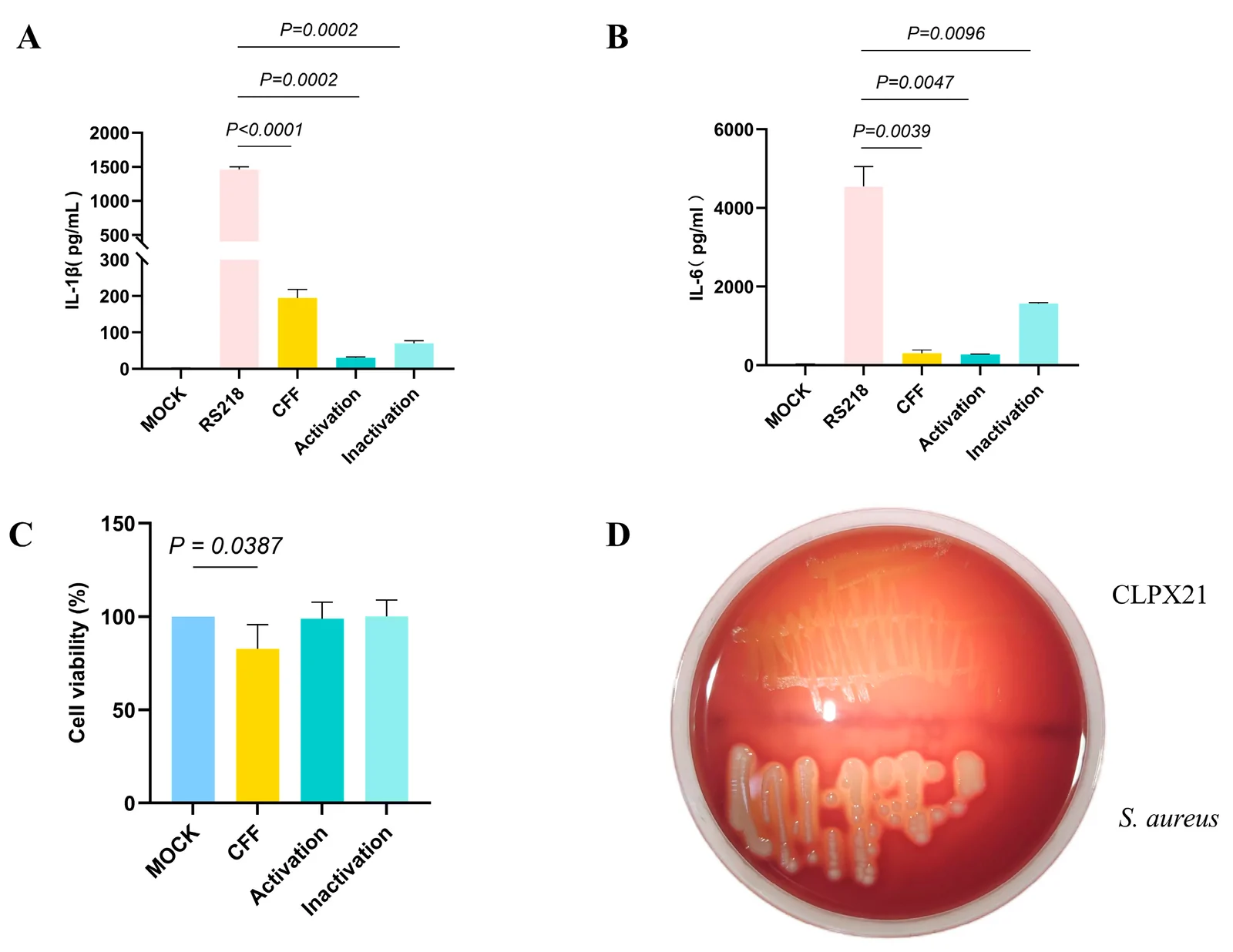
Anti-inflammatory activity of CLPX21 and its safety evaluation in vitro. (**A**) IL-1β secretion level in mice primary peritoneal macrophages. (**B**) IL-6 secretion in mice primary peritoneal macrophages. (**C**) Cytotoxicity of CLPX21 CFF, activation WCS, and inactivation WCS. (**D**) Hemolytic activity.

The anti-inflammatory properties of CLPX21 were investigated in *E. coli*-infected mice primary macrophages. To investigate whether the anti-inflammatory activity was attributable to metabolites, activation WCS, or inactivation WCS of CLPX21, different forms of the strain were used. The results showed that *E. coli* RS218 infection induced high production levels of inflammatory cytokines including IL-1β and IL-6, whereas the CFF, activation WCS, and inactivation WCS significantly inhibited *E. coli*-induced production of inflammatory cytokines (Figure 3A,B). Particularly, the activation WCS almost completely abrogated this inflammatory response, indicating that CLPX21 produces anti-inflammatory metabolites. The results demonstrate that CLPX21 exhibits potent anti-inflammatory activity.

### 3.4. Safety Evaluation of CLPX21 In Vitro

To investigate the safety of CLPX21 in vitro, mice macrophage viability was investigated using trypan blue assays and hemolytic activity. The results showed that the CFF exhibited cytotoxicity to some extent, with cell viability decreasing to 82.73%, while the activation WCS and inactivation WCS exhibited no effect on cell viability (Figure 3C). Furthermore, CLPX21 exhibited non-hemolytic activity on blood agar plates, but the positive control *S. aureus* clearly exhibited hemolytic activity (Figure 3D).

### 3.5. Genomic Analysis of CLPX21

**Figure 4:**
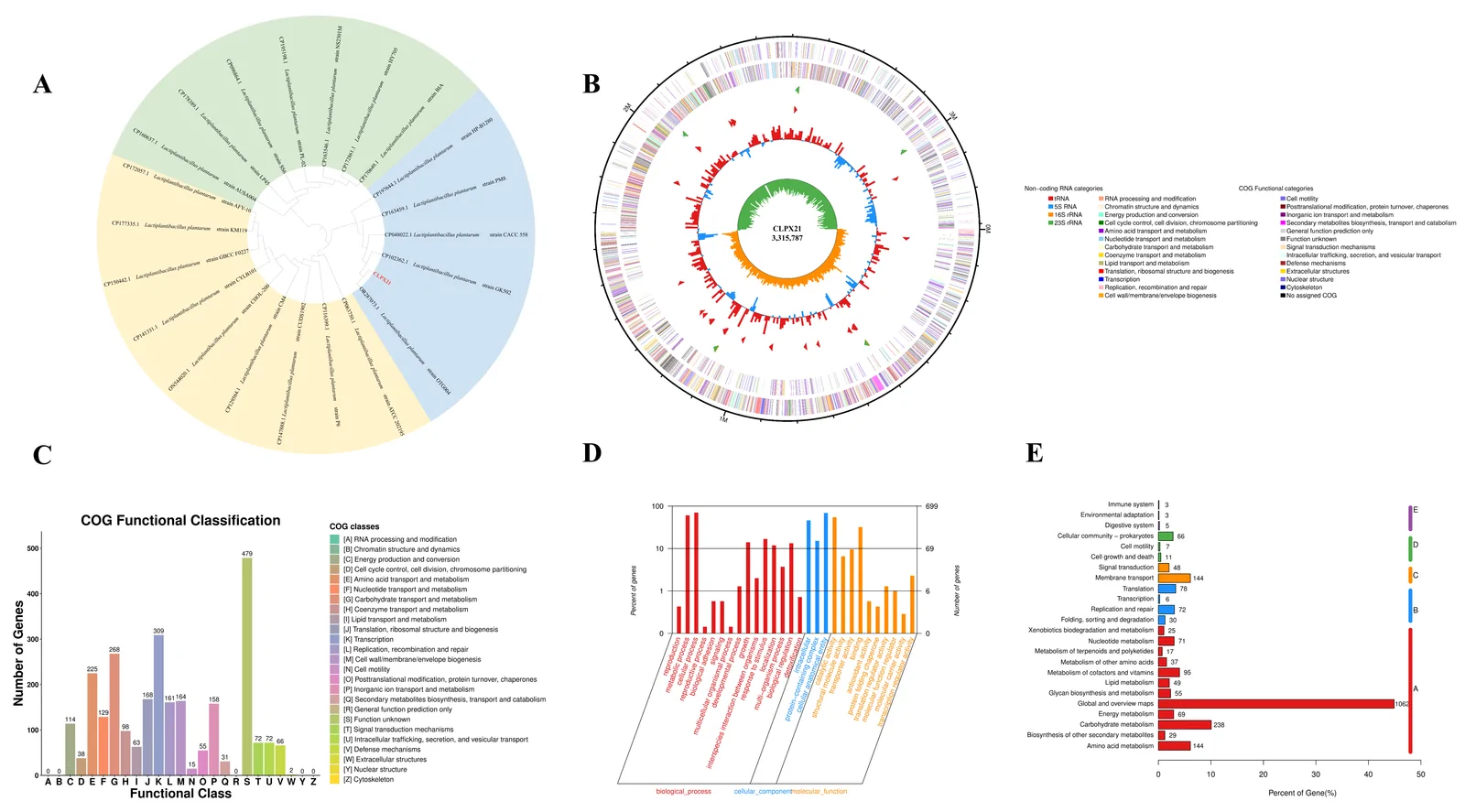
Genome properties and functional annotation of CLPX21. (**A**) Phylogenetic analysis of CLPX21. (**B**) Circular genome map of CLPX21. (**C**) Functional category distribution of annotated genes in COG database. (**D**) Functional term distribution of annotated genes in GO database. (**E**) Functional pathway distribution of annotated genes in KEGG database.

Phylogenetic analysis based on 16S rRNA gene sequences confirmed the taxonomic identity of CLPX21, which clustered closely with *Lactiplantibacillus plantarum* reference strains (Figure 4A).

Genome sequencing analysis indicates that the CLPX21 genome comprises a chromosome of 3,315,787 bp (Figure 4B), with a guanine–cytosine (GC) content of 45.3%, and one plasmid (11, 337 bp) (Table S2). In addition, the ncRNA in the CLPX21 genome comprises six 5S rRNA, five 16S rRNA, five 23S rRNA, and 72 tRNA genes (Table S2). Furthermore, the CLPX21 chromosome is predicted to contain eight prophages (Table S2). Based on COG functional classification analysis, annotated genes in CLPX21 were enriched in categories related to transcription, translation, ribosomal function, and amino acid and carbohydrate transport and metabolism, reflecting active metabolism and environmental adaptation potential (Figure 4C). Gene ontology (GO) functional classification analysis further revealed that annotated genes were enriched in terms of biological processes, cellular components, and molecular functions, mainly related to metabolic processes, cellular processes, growth, responses to stimuli, catalytic activity, antioxidant activity, transcription, and translation regulator activity et al. (Figure 4D). KEGG functional classification analysis showed that the annotated genes were mainly enriched in amino acid metabolism, carbohydrate metabolism, nucleotide metabolism, and membrane transport et al. (Figure 4E).

**Figure 5:**
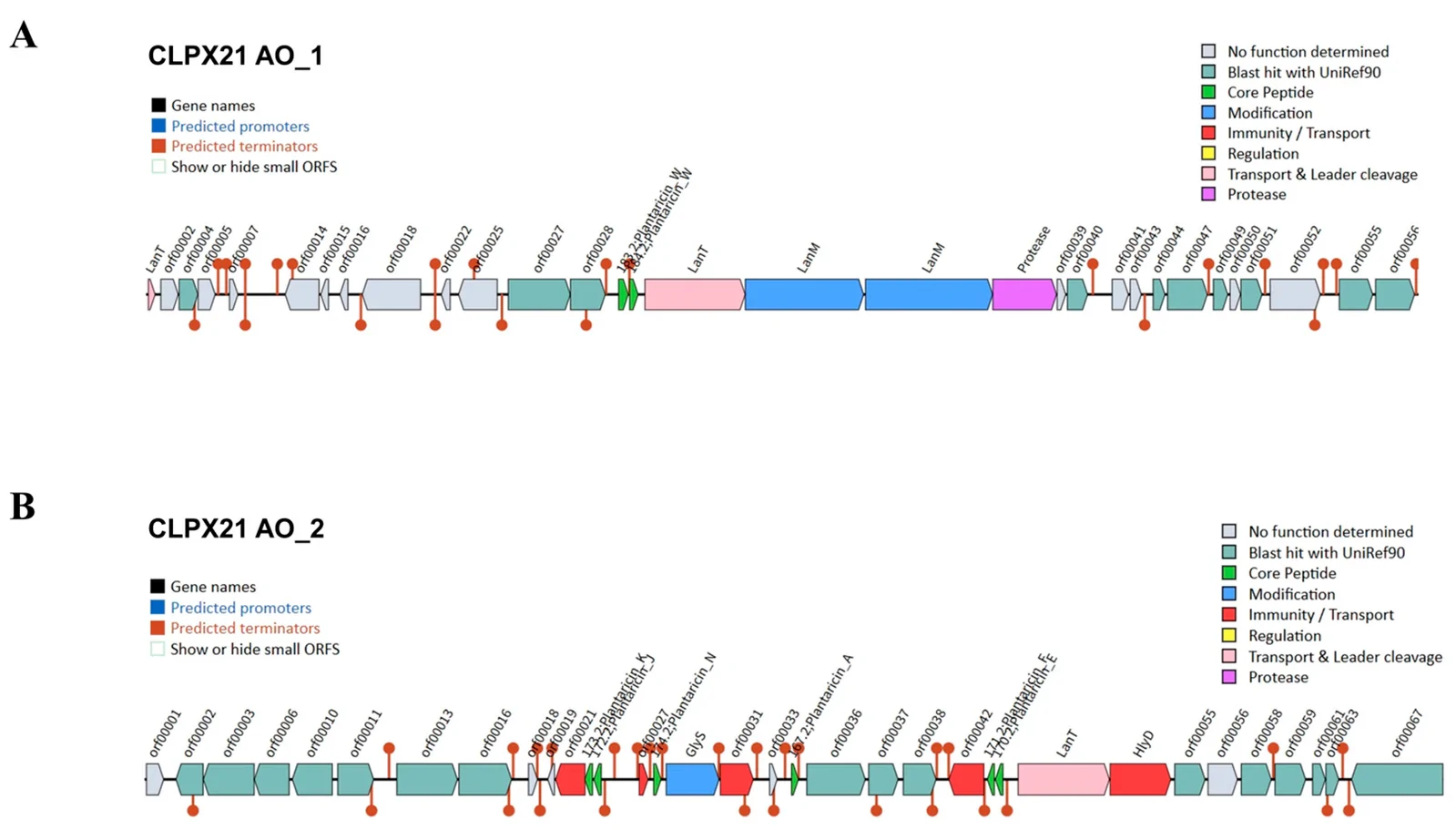
Predicted bacteriocin-producing gene clusters in CLPX21. (**A**) Bacteriocin-producing gene cluster AO_1. (**B**) Bacteriocin-producing gene cluster AO_2. The schematic representation shows the arrangement of ORFs within the cluster, with predicted promoters, terminators, and functional modules indicated by different colors. Core peptides, modification enzymes, immunity proteins, regulatory elements, and transport-related genes are highlighted.

Interestingly, two complete bacteriocin gene clusters (named AO_1 and AO_2) were identified in the CLPX21 genome, with sizes of 27 kb (containing 56 open reading frames) and 29 kb (containing 67 open reading frames), respectively (Figure 5A,B). Functional annotation reveals that the AO_1 gene cluster contained genes encoding core peptides, modifying enzymes, transport and leader cleavage proteins, regulatory elements, and secretion-associated proteins. Its core region included genes highly homologous to the plantaricin family bacteriocin PlnW (Table S3). Simultaneously, orf00001 and orf00033 were annotated as transporters and leader cleavage enzymes, demonstrating that this strain possesses the abilities of transmembrane transport and efficient secretion of mature bacteriocins. The AO_2 gene cluster contained genes encoding core peptides, modifier enzymes, immunomodulators/transporters, regulatory proteins, and secretory proteins (Figure 5B). Multiple genes highly similar to the plantaricin family (e.g., PlnA, PlnJ, PlnK, PlnN, PlnE, and PlnF) were detected in the core region (Table S4), confirming that CLPX21 possesses the genetic property to produce Class II peptide bacteriocins. Furthermore, genes including orf00021, orf00027, orf00031, and orf00042 were annotated as immunity proteins, indicating that this strain exhibits a self-protection system against the cytotoxic effects of endogenous antimicrobial agents.

Notably, gene clusters associated with secondary metabolite biosynthesis were identified in CLPX21 (Table S5). These gene clusters included RiPPs, Lanthipeptide-class-II, NRPS and NRPS-like, terpenes, T3PKS, and lactone autoinducers (Table S5), suggesting that CLPX21 not only has the potential to produce bioactive compounds with antibacterial, antioxidant, anti-inflammatory, anticancer, and quorum-sensing activities, but also possesses strong environmental adaptability and the ability to establish a competitive advantage through chemical defense within the complex gut ecosystem.

VFDB annotation results indicated that the CLPX21 genome harbored seven virulence-associated genes, annotated as *hasC, clpP, eno, rfbA, rfbB, lisR*, and *tufA* with below 80% identity (Table S6). However, according to EFSA, genes with above 80% sequence identity are considered virulence factors [15]. In addition, these genes’ functions are related to basal metabolism and cellular homeostasis, such as carbohydrate metabolism, stress tolerance, regulatory responses, adhesion, immunomodulation, and surface interactions. These functions are highly conserved within *Lactobacillus* species, primarily supporting survival and colonization under harsh intestinal conditions like low pH and high osmotic pressure, rather than mediating typical pathogenic mechanisms such as invasion, toxin secretion, or immune evasion.

## 4. Discussion

LAB have been shown to be beneficial for host health with their multiple functions, such as antimicrobial activity, anti-oxidative activity, anti-inflammatory activity, and regulating gut microbiota homeostasis [16]. Therefore, LAB have been widely used to treat infectious and non-infectious diseases and enhance host immunity. Our study identified and characterized a new LAB strain, *L. plantarum* CLPX21, isolated from fermented vegetables through whole-genomic analysis and basic probiotic property assays in vitro. Our results have shown that CLPX21 exhibited potent antimicrobial activity against foodborne pathogenic bacteria and anti-inflammatory activity. These results indicate that CLPX21 could be a potential probiotic.

Tolerance to the harsh environment of host gastrointestinal tracts, including acid and bile salt tolerance, is the primary criterion for the selection of potential probiotics. It has been widely reported that *L. plantarum* strains exhibit acid and bile salt tolerance to some extent. Hu et al. reported that *L. plantarum* ZYN-04 isolated from traditional fermented shredded potato showed high survival rates at low pH and under the treatment of bile salt for 4 h, where their viable counts were above 3.2 and 3.5 log CFU/mL, respectively [17]. Liao et al. reported that *L. plantarum* NCU001697 from fermented green tea shows low survival at pH = 2 for 3 h, with only a viable count of 1.86 log CFU/mL, but shows high survival in the presence of bile salts (0.4%) for 3 h, with a viable count of 5.04 log CFU/mL [18]. Our study showed that *L. plantarum* CLPX21 exhibits excellent survival in low-pH conditions and various bile salt concentrations, with survival rates approaching nearly 100%. This robust gastrointestinal stress resistance is consistent with our whole-genome sequencing analysis, which identified the expression of stress tolerance-related genes *clpP* and *lisR* [19,20].

To exert a beneficial effect on the host, LAB need to colonize and persist in the gastrointestinal tract, so adhesion capacity is another important probiotic selection criterion. Our whole-genome sequencing analysis annotated several adhesion-related genes, including *tufA*, *hasC*, *rfbA*, *rfbB*, and *eno*. CLPX21 exhibited potent adhesion capacity, as evidenced by its 47.15% auto-aggregation, high co-aggregation rates with *E. coli* (70.14%), *Salmonella* (68.43%), and *S. aureus* (67.77%), and 6.37% cell adherence rate towards IPEC-J2. Notably, among these adhesion-related genes, *tufA* encodes elongation factor Tu (EF-Tu) that acts as a direct adhesin to mediate specific binding to mucins, fibronectin, and intestinal epithelial cells [21]. Similarly, *eno* encodes another adhesin that reinforces attachment to host extracellular matrix components [22,23]. In contrast, *hasC*, *rfbA*, and *rfbB* are not classical adhesion genes and do not directly encode fimbriae or adhesins, but participate in the biosynthesis of cell-wall polysaccharides and exopolysaccharides (EPSs), which modulate surface hydrophobicity and cell envelope stability [24,25]. Consistently, *L. plantarum* has been reported to adhere to various epithelial cell types, including Caco-2 cells, with adhesion capacity being positively associated with auto-aggregation and cell surface hydrophobicity [26]. These genes together contribute to the aggregation and epithelial adhesion phenotypes of CLPX21, highlighting its robust gut colonization potential. However, these genes exert different mechanisms to modulate LAB adhesion and the exact mechanism of adhesion of CLPX21 needs to be further studied.

LAB strains often produce antimicrobial substances, such as organic acids, hydrogen peroxide, acetyl-aldehyde, and carbon dioxide, as well as bacteriocins. Multiple studies have successfully identified various bacteriocins with distinct antimicrobial activities from different *L. plantarum* strains, such as plantaricin MG, plantaricin ZJ5, bacteriocins ST28MS and ST26MS, bacteriocin BacTN635, and bacteriocin Lac-B23 [27,28,29,30]. These bacteriocins are predominantly peptides with antimicrobial activity against pathogens. A recent study has reported that a novel antimicrobial nanobacteriocin derived from *L. plantarum* strains exhibits broad-spectrum antibacterial activity against *Listeria monocytogenes*, *Acinetobacter baumannii*, and *Vibrio parahaemolyticus* [31]. These discoveries not only enrich the antimicrobial peptide resource library but also provide new directions for the development of *L. plantarum*. Thus, *L. plantarum* serves as an important reservoir for the discovery of novel antimicrobial peptides. Our whole-genome analysis indicates that CLPX21 carries two bacteriocin biosynthesis gene clusters, suggesting its antibacterial effect is likely related to the production of bacteriocin-like substances. In vitro anti-infective assays showed that CLPX21 had potent antimicrobial activity against *E. coli*, *S. aureus*, and *Salmonella*. Additionally, CLPX21 inhibited biofilm formation. Notably, an acidic environment at pH = 4 did not exhibit antibacterial or anti-biofilm effects compared with CLPX21 CFF, suggesting that the antimicrobial activity of CLPX21 can not entirely be attributed to organic acid production, but may be attributed to the combined effects of bacteriocin-mediated bactericidal activity, disruption of bacterial quorum sensing, and interference with extracellular matrix synthesis. These results demonstrate that CLPX21 has promising potential to be developed as a functional probiotic for controlling pathogenic bacterial infections and reducing biofilm-related risks. Accordingly, its anti-infective efficacy in vivo warrants further investigation in future studies.

Besides direct antimicrobial activity, *L. plantarum* strains also exert anti-infective effects through modulating the host inflammatory response. Inhibiting excessive inflammation represents a key strategy to alleviate pathogen-induced tissue damage. *L.* *plantarum* strains have been reported to inhibit pathogen- and LPS-induced inflammatory response through suppressing the activation of the key NF-κB and MAPK inflammatory signaling pathways as well as downregulating the expression of critical inflammatory factors, such as TLR4, MyD88, IL-6, and TNF-α [7,32,33,34,35]. Interestingly, Wu et al. reported [32] that an *L. plantarum* strain inhibited *Salmonella*-induced NLRP3 inflammasome activation through activating autophagy, indicating that LAB exert multi-signal regulatory effects on the host against pathogenic infections. Although our study did not investigate the inhibitory effect of CLPX21 on inflammatory signaling pathways including NF-κB, MAPK, and NLRP3, both the CFF and live WCS of CLPX21 significantly suppressed *E. coli*-induced IL-6 and IL-1β secretion in macrophages, suggesting that CLPX21 possesses potent anti-inflammatory activity. Notably, this anti-inflammatory outcome could be partially attributed to the direct bactericidal capacity of both the CFF and live CLPX21 against RS218, which reduces the number of invasive pathogens, thereby attenuating inflammatory signaling activation, consistent with our time–killing and anti-biofilm data. Furthermore, CLPX21 may alter macrophage phagocytic efficiency toward RS218. It has been reported that LAB can enhance the phagocytic and killing capabilities of macrophages towards bacteria [36,37]. However, the anti-inflammatory effect of its CFF alone indicates that CLPX21 secretes bioactive metabolites with anti-inflammatory activity consistent with genomic analysis data, which is partially independent of the effect of phagocytosis efficiency. Therefore, the exact anti-inflammatory effect and mechanism in vivo need to be further studied.

## 5. Conclusions

In conclusion, *L. plantarum* CLPX21 exhibits promising probiotic potential with multiple beneficial functions. Genomic analysis and in vitro assays collectively demonstrate that CLPX21 exhibits potent stress tolerance capacity, adhesion capacity, and antimicrobial activity. Additionally, CLPX21 effectively suppresses pro-inflammatory cytokine secretion, indicating its immunomodulatory activity. Notably, safety evaluations confirm that CLPX21 does not exhibit hemolytic activity. These findings suggest that CLPX21 has great potential to be developed as a promising candidate probiotic for controlling pathogenic infection and modulating host immune responses. However, the in vivo efficacy of CLPX21 and its specific molecular mechanisms need to be further studied for its application.

## Data Availability

All data are available within the article and from the corresponding author upon reasonable request. The genomic analysis data have been deposited in the NCBI SRA database under accession number SRR37824858 for CLPX21. The data are publicly accessible at https://trace.ncbi.nlm.nih.gov/Traces/?view=run_browser&acc=SRR37824858&display=metadata. The data was accessed on 2 July 2026.

## Supplementary Materials

The following supporting information can be downloaded at https://www.mdpi.com/article/10.3390/microorganisms14081789/s1. Table S1: The adhesion rate of CLPX21 towards IPEC-J2 cells. Table S2: General genome information of CLPX21. Table S3: Functional annotation of predicted bacteriocin-related genes in CLPX21 AO_1. Table S4: Functional annotation of predicted bacteriocin-related genes in CLPX21 AO_2. Table S5: Predicted secondary metabolite biosynthesis gene clusters in CLPX21. Table S6: Predicted virulence-associated genes identified in the genome of CLPX21.
