# Supplementary material for: Whole-Genomic and Functional Characterization of Lactiplantibacillus plantarum CLPX21 as Potent Probiotic Candidate
Source: Microorganisms. 2026 Aug 14;14(8):1789. doi: 10.3390/microorganisms14081789 (PMC13515879; doi:10.3390/microorganisms14081789)
Supplement: Supplementary file 1 [file microorganisms-14-01789-s001.zip › microorganisms-4458768-supplementary.pdf]

Table S1. The adhesion rate of CLPX21 towards IPEC-J2 cells

L. plantarum	Adhesion rate
CLPX21	6.37%

Table S2. General genome information of CLPX21

Indicator	Number or content
Chromosome	3,315,787-bp
Plasmid	1
GC content of chromosome	45.3%
Coding genes number	3176
Total length of coding genes	2,808,984-bp
23S rRNA	5
16S rRNA	5
5S rRNA	6
tRNA	72
CRISPR	0
Prophage	8

Table S3. Functional annotation of predicted bacteriocin-related genes in CLPX21 AO_1

ORF	Gene Strand	Gene Length (bp)	Function
orf00001	+	141	AbpT bacteriocin export accessory protein
orf00002	+	366	/
orf00004	+	396	Protein MesC OS
orf00005	+	360	/
orf00007	+	177	/
orf00014	-	714	/
orf00015	-	162	/
orf00016	-	168	/
orf00018	-	1233	/
orf00022	-	186	/
orf00025	-	819	/
orf00027	+	1311	Bacteriocin production related histidine kinase
orf00028	+	735	response regulator

orf00029	+	201	Plantaricin_W_beta183.2; Plantaricin_W
orf00030	+	177	Plantaricin_W_alfa184.2; Plantaricin_W
orf00033	+	2118	Lantibiotic mersacidin transporter system
orf00034	+	2502	Lantibiotic mersacidin modifying enzyme
orf00036	+	2688	Lantibiotic modifying enzyme
orf00038	+	1347	Epidermin leader peptide-processing serine protease
orf00039	+	180	/
orf00040	+	426	Bacteriocin transport accessory protein, putative
orf00041	+	342	/
orf00043	+	234	/
orf00044	+	249	Transposase
orf00047	+	840	Insertion element
orf00049	+	303	Transcriptional repressor
orf00050	+	219	/
orf00051	+	444	NADH pyrophosphatase
orf00052	+	1059	/
orf00055	+	705	Insertion element
orf00056	+	822	Uncharacterized transposase-like protein

Table S4. Functional annotation of predicted bacteriocin-related genes in CLPX21 AO_2

ORF	Gene Strand	Gene Length (bp)	Function
orf00001	+	390	/
orf00002	-	615	Galactoside O-acetyltransferase
orf00003	-	1152	Methylenomycin A resistance protein
orf00006	-	789	5-phospho-D-ribitylamino
orf00010	-	906	HTH-type transcriptional activator RhaR
orf00011	+	813	Sugar phosphatase YidA
orf00013	+	1374	Branched-chain amino acid transport system carrier protein
orf00016	+	1194	Na(+)/H(+) antiporter
orf00018	+	201	/
orf00019	-	150	/
orf00021	-	666	Putative bacteriocin Immunity protein
orf00022	-	171	ComC; Bacteriocin_IIc; 173.2; Plantaricin_K
orf00023	-	165	172.2; Plantaricin_J
orf00027	+	198	Immunity protein PlnM
orf00028	+	165	Bacteriocin_IIc; 174.2; Plantaricin_N
orf00029	+	1197	PlnO
orf00031	+	744	Immunity protein PlnI
orf00033	+	171	/

orf00034	+	153	Antimicrobial17; Bacteriocin_IIc; 167.2; Plantaricin_A
orf00036	+	1326	Bacteriocin production related histidine kinase
orf00037	+	666	Response regulator PlnC, activator
orf00038	+	741	Response regulator PlnD
orf00042	-	789	Immunity protein PlnI
orf00045	-	156	ggmotif; Lactococcin; Bacteriocin_IIc; 171.2; Plantaricin_F
orf00047	-	168	170.2; Plantaricin_E
orf00050	+	2070	Bacteriocin ABC-transporter, ATP-binding and permease protein PlnG
orf00053	+	1374	Accessory factor for ABC-transporter PlnH
orf00055	+	687	PlnS
orf00056	+	666	/
orf00058	+	678	PlnS
orf00059	+	693	PlnS
orf00061	+	288	Toxin HigB-1
orf00063	+	291	PlnY
orf00067	-	2073	DNA helicase IV

Table S5. Predicted secondary metabolite biosynthesis gene clusters in CLPX21

Location	Region	Type	From	To
chromosome	Region 1	RiPP-like	380,105	392,177
chromosome	Region 2	NRPS, NRPS-like	519,983	579,666
chromosome	Region 3	terpene-precursor	1,403,370	1,424,260
chromosome	Region 4	RiPP-like	1,593,593	1,605,749
chromosome	Region 5	lanthipeptide-class-ii	1,605,778	1,628,468
chromosome	Region 6	T3PKS	1,828,510	1,869,679
chromosome	Region 7	terpene	2,938,826	2,959,707
chromosome	Region 8	cyclic-lactone-autoinducer	3,220,524	3,241,229

Table S6. Predicted virulence-associated genes identified in the genome of CLPX21

VFid	Function	SubType	Type
VFG005871	UTP--glucose-1-phosphate uridylyltransferase HasC	hasC	Immune modulation
VFG000077	ATP-dependent Clp protease proteolytic subunit	clpP	Stress survival
VFG005582	phosphopyruvate hydratase	eno	Exoenzyme
VFG005898	glucose-1-phosphate	rfbA	Immune modulation

	thymidylyltransferase RfbA		
VFG006022	dTDP-glucose 4,6-dehydratase	rfbB	Immune modulation
VFG006826	two-component response regulator	lisR	Regulation
VFG046474	elongation factor Tu	tufA	Adherence
